# Utility of the Rio Score and Modified Rio Score in Korean Patients with Multiple Sclerosis

**DOI:** 10.1371/journal.pone.0129243

**Published:** 2015-05-26

**Authors:** Jae-Won Hyun, Su-Hyun Kim, In Hye Jeong, Suk-Won Ahn, So-Young Huh, Min Su Park, Young In Eom, In Soo Joo, Joong-Yang Cho, Eun Bin Cho, Ju-Hong Min, Byoung Joon Kim, Nam-Hee Kim, Jeeyoung Oh, Kee Duk Park, Ho Jin Kim

**Affiliations:** 1 Department of Neurology, Research Institute and Hospital of National Cancer Center, Goyang, Korea; 2 Chung-Ang University, College of Medicine, Seoul, Korea; 3 Kosin University, College of Medicine, Busan, Korea; 4 Yeungnam University, College of Medicine, Daegu, Korea; 5 Ajou University, School of Medicine, Suwon, Korea; 6 Ilsan Paik Hospital, Inje University, College of Medicine, Goyang, Korea; 7 Samsung Medical Center, Sungkyunkwan University, School of Medicine, Seoul, Korea; 8 Dongguk University Ilsan Hospital, Goyang, Korea; 9 Konkuk University, School of Medicine, Seoul, Korea; 10 Ewha Womans University, Graduate School of Medicine, Seoul, Korea; 11 Neuroscience Center, Samsung Medical Center, Seoul, Korea; University of Düsseldorf, GERMANY

## Abstract

**Objectives:**

Early identification of suboptimal responders to multiple sclerosis (MS) treatment is critical for optimizing therapeutic decisions. The Rio score (RS) and modified Rio score (MRS) were developed to discriminate the responses to interferon-beta (IFNB) treatment in MS patients. This study was performed to evaluate the utility of RS and MRS in daily clinical practice in Korea.

**Methods:**

This was a real-world setting, multicenter, retrospective study of MS patients treated with IFNB from 10 hospitals in Korea. We investigated whether the RS and MRS at the early stage of IFNB therapy could predict treatment responses over 3 years. Suboptimal treatment responses at 3 years were defined as the presence of clinical relapse and/or EDSS progression and/or patients who had been treated with INFB for at least for 1 year and therapy was switched due to perceived treatment failure during the 2 years of follow-up.

**Results:**

Seventy patients (50 females and 20 males) were enrolled; 92% (12/13) of patients with high RS and 86% (12/14) of patients with high MRS (score 2 or 3) were suboptimal responders, whereas 93% (53/57) of patients with low RS and 93% (52/56) patients with low MRS (score 0 or 1) showed optimal responses. New active lesions on MRI with clinical relapse in high RS and MRS were the most common combination in suboptimal responders.

**Conclusions:**

We confirmed that RS and MRS at 6–15 months of IFNB therapy were useful for predicting poor responders over 3 years.

## Introduction

Interferon-beta (IFNB) is the established first-line therapy for relapsing-remitting multiple sclerosis (MS) [1]. However, the individual response to interferon-beta is heterogeneous [2]. There is now a broad range of treatment options available for MS [3], and it becomes imperative to identify suboptimal responders to first-line therapy early during the course of treatment to optimize therapeutic decisions [4,5]. Clinical and MRI measures have proven helpful in detecting poor-responders among MS patients during early in the course of treatment with IFNB [6].

The Rio score (RS) is a recently developed scoring system that consists of a combination of clinical and MRI parameters to predict suboptimal responders [7]. The Modified Rio score (MRS) is a simplified version of RS, which excludes expanded disability status scale (EDSS) progression and modified items of relapses and MRI lesions [8]. These scores were estimated after 1 year of IFNB therapy with the aim of identifying patients that will have ongoing disease activity and become suboptimal responders in the ensuing 2 years.

Previous studies in Asia demonstrated that, after careful exclusion of neuromyelitis optica spectrum disorder (NMOSD), the therapeutic response to IFNB does not differ fundamentally between Asian and Caucasian populations with MS [9–11]. Furthermore, we showed recently that the McDonald criteria for diagnosis of MS [12,13] were also suitable for evaluation of Korean MS patients [9,14]. Therefore, therapeutic monitoring of IFNB in MS patients using RS and MRS would be also helpful to evaluate Asian MS populations, but the previous validation of RS and MRS was performed only in Western MS patients based mainly on a prospective research cohort [7,8]. The present study was performed to evaluate the utility of RS and MRS in daily clinical practice in a Korean multicenter cohort.

## Methods

### Ethics Statement

The Institutional Review Board of National Cancer Center approved the study protocol (NCC2014-0046) and waived the requirement for informed consent due to the use of de-identified data.

### Study design and patients

This was a real-world setting, multicenter, retrospective study of MS patients treated with IFNB. Patients were recruited from 10 referral hospitals in Korea. Patients with relapsing MS treated with IFNB over 1 year with a follow-up duration of least 3 years with adequate medical records and MRI scans were enrolled in this study [12,13]. Due to the retrospective nature of this study, patients who had a baseline and follow-up brain MRI after 6–15 months of IFNB (not confined to the patients who had a follow-up MRI after 12 months) were included in the cohort. Patients who had received other disease modifying treatments for MS before IFNB were excluded.

### Defining scoring systems and risk groups

RS and MRS were defined as described previously (Table 1) [7,8]. RS and MRS were not confined after 12 months but after 6–15 months of IFNB therapy according to the follow-up duration of MRI. According to the scores, patients were divided into a low-risk group (score 0 or 1), or high-risk group (score 2 or 3) for a suboptimal response to treatment after 6–15 months of INFB. To decrease the inter-observer variability, a web-based central database for MRI was established and MRI scans were independently analyzed by two neurologists.

**Table 1:** Rio and modified Rio scores (assessed at the first year of interferon therapy) [7,8].

Rio score	
MRI criterion = 1	if the patient had (on the yearly MRI scan) > 2 active T2 lesions, defined as new or enlarging T2-weighted lesions, plus the number of gadolinium-enhancing (Gd) T1-weighted lesions over the first year
Relapse criterion = 1	if the patient experienced ≥ 1 relapse over the first year
EDSS criterion = 1	if there was an increase in the patient’s EDSS score of ≥1 point, sustained over at least 6 months and confirmed at the end of the follow-up period.
*The sum of these three criteria classifies patients into those having a score of 0*, *1*, *2 or 3*	
**Modified Rio score**	
MRI criterion = 1	if the patient has had > 4 new T2 lesions
Relapse criterion = 1	if the patient experienced 1 relapse
Relapse criterion = 2	if the patient experienced ≥ 2 relapses
Score = 0	if new T2 lesions ≤ 4 and relapses = 0
Score = 1	if new T2 lesions ≤ 4 and relapses = 1; or new T2 lesions >4 and relapses = 0
Score = 2	if new T2 lesions ≤ 4 and relapses ≥ 2; or new T2 lesions >4 and relapses = 1
Score = 3	if new T2 lesions > 4 and relapses ≥ 2

### Outcomes

Suboptimal responses at 3 years were defined as the presence of clinical relapse (accompanied by an appropriate new neurological abnormality; lasting at least 24 hours in the absence of fever; and preceded by stability or improvement for at least 30 days) and/or 6 months confirmed EDSS progression (1 point for patients with 1-year EDSS < 6, 0.5 points for EDSS ≥ 6) during the ensuing 2 years of IFNB therapy [1,7,8]. Due to the retrospective nature of this study, if we had used the previous definition of suboptimal responders, the sensitivities of RS and MRS would have been too narrow to evaluate suboptimal responders in daily practice. Therefore, we also included nine patients who had been treated with INFB for at least for 1 year and therapy was switched due to perceived treatment failure (clinical relapses) during the 2 years of follow-up, as suboptimal responders to IFNB. In addition, we examined whether the high-risk groups of RS and MRS became suboptimal responders and RS and MRS were well matched.

### Statistical analysis

Comparisons of patients in the high- and low-risk groups were performed by the chi-squares test, or Fisher’s exact test for categorical data.

## Results

A total of 70 MS patients treated with IFNB from 10 centers was enrolled in this study. Among the 70 patients, 50 were women and 20 were men. The mean age of onset was 29.5 ± 9.6 years and mean follow-up duration was 6.5 ± 2.8 years. The median baseline EDSS score at commencement of IFNB treatment was 2 (range 0–5) and the median interval from onset to IFNB therapy was 9 (range 1–92) months. (Table 2)

**Table 2:** The demographics of the patients (Total n = 70).

Gender (male:female)	20:50
Mean age of onset (years, mean (SD^1^))	29.5 (9.6) (range 12–51)
Mean follow-up duration (years, mean (SD))	6.5 (2.8) (range 2–14)
Median EDSS^2^ score at the starting Interferon therapy	2 (range 0–5, IQR^3^ 1–3)
Median delay from onset to Interferon therapy (months)	9 (range 1–92, IQR 4–25)

Abbreviation: ^1^SD = standard deviation, ^2^EDSS = expanded disability status scale, ^3^IQR = interquartile range

First, sixteen (23%) of the seventy patients were classified as suboptimal responders after 3 years of IFNB therapy and high-risk groups of RS and MRS predicted suboptimal treatment response well. The high-risk group of RS consisted of 13 (19%) patients and the low-risk group consisted of 57 (81%). Similarly, 14 of 70 (20%) patients were included in the high-risk group of MRS and the low-risk group of MRS consisted of 56 (80%) patients. Twelve of thirteen (92%) patients in the high-risk group of RS and 12 of 14 (86%) patients in the high-risk group of MRS showed suboptimal responses. In contrast, 53 of 57 (93%) patients in the low-risk group of RS and 52 of 56 (93%) patients in the low-risk group of MRS were optimal responders. There was a significant difference between the high and low score groups in terms of predicting suboptimal responses (p < 0.001)(Table 3). In addition, the high-risk groups of RS and MRS showed high sensitivity (both 75%) and specificity (98%, 96%, respectively) in terms of predicting suboptimal responders (Table 4). The combination of new active lesions on follow-up MRI and relapses in RS as well as MRS were the most common combinations of suboptimal responders (Table 5).

**Table 3:** Scoring values of Rio (RS) and modified Rio (MRS) scores.

Rio score	Optimal responders	Suboptimal responders	Total n = 70
**0**	37/40 (92%)	3/40 (8%)	40 (57%)
**1**	16/17 (94%)	1/17 (6%)	17 (24%)
**2**	1/9 (11%)	8/9 (89%)	9 (13%)
**3**	0/4 (0%)	4/4 (100%)	4 (6%)
**Rio score**	Optimal responders	Suboptimal responders	p-value
Low RS (0 or 1)	53	4	<0.001
High RS (2 or 3)	1	12	<0.001
**Modified Rio score**	Optimal responders	Suboptimal responders	Total n = 70
**0**	39/42 (93%)	3/42 (7%)	42 (60%)
**1**	13/14 (93%)	1/14 (7%)	14 (20%)
**2**	2/12 (7%)	10/12 (83%)	12 (17%)
**3**	0/2 (0%)	2/2 (100%)	2 (3%)
**Modified Rio score**	Optimal responders	Suboptimal responders	p-value
Low MRS (0 or 1)	52	4	<0.001
High MRS (2 or 3)	2	12	<0.001

**Table 4:** Statistical values of Rio (RS) and modified Rio (MRS) scores.

Rio score (RS)			
Low risk group of RS for optimal response		High risk group of RS for suboptimal response	
Sensitivity	98%	Sensitivity	75%
Specificity	75%	Specificity	98%
Accuracy	93%	Accuracy	93%
Positive predictive value	93%	Positive predictive value	92%
Negative predictive value	92%	Negative predictive value	93%
**Modified Rio score (MRS)**			
Low risk group of MRS for optimal response		High risk group of MRS for suboptimal response	
Sensitivity	96%	Sensitivity	75%
Specificity	75%	Specificity	96%
Accuracy	91%	Accuracy	91%
Positive predictive value	93%	Positive predictive value	86%
Negative predictive value	86%	Negative predictive value	93%

**Table 5:** The combinations of components of Rio (RS) and modified Rio (MRS) scores of the suboptimal responders (Total n = 16).

Combination of Rio score	n(%)
MRI(-) Relapse(-) EDSS(-)	3 (19)
MRI(-) Relapse(+) EDSS(-)	1 (6)
MRI(+) Relapse(+) EDSS(-)	8 (50)
MRI(+) Relapse(+) EDSS(+)	4 (25)
**Combination of modified Rio score**	
MRI(-) Relapse 0	3 (19)
MRI(-) Relapse 1	1 (6)
MRI(-) Relapse 2	4 (25)
MRI(+) Relapse 1	5 (31)
MRI(+) Relapse 2	3 (19)

**Fig 1:**
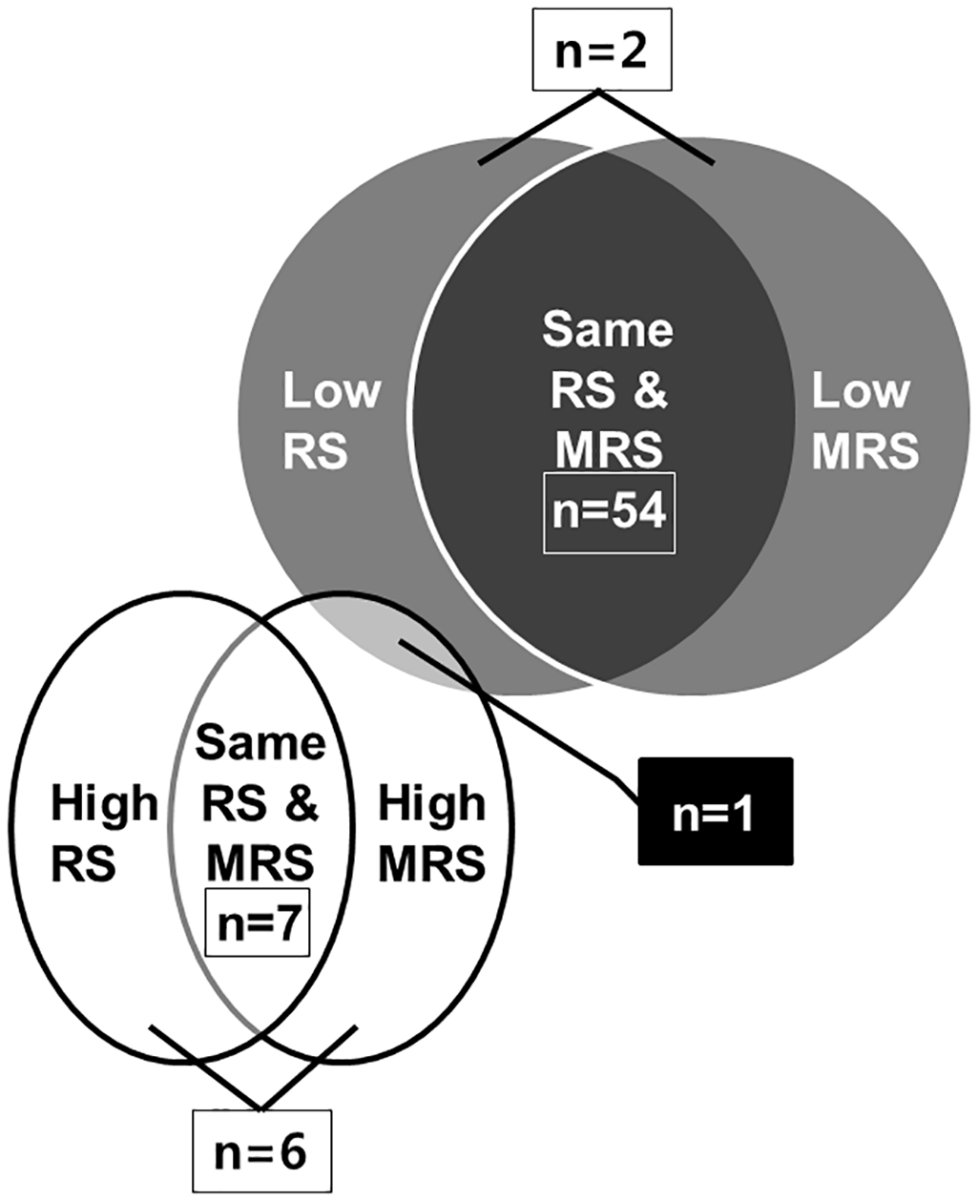
Comparison of the high and low risk groups of Rio and modified Rio scores.

Second, RS and MRS were well matched in high- or low-risk groups, respectively (Fig 1). Sixty-one of the seventy (87%) patients had the same scores on RS and MRS. RS and MRS differed in nine patients, but there was no overlap between the low- and high-risk groups of RS and MRS except one case (1%) with a mixed low-risk group of RS and high-risk group of MRS. The patient who had different RS and MRS experienced two clinical relapses during 1 year of IFNB with two new active lesions on follow-up MRI scan, and therefore RS was 1 but MRS was estimated as 2; the patient finally became an optimal responder.

## Discussion

We demonstrated that RS and MRS measured at 6–15 months of IFNB therapy were good predictors of treatment response in the ensuing 2 years in Korean MS patients. Despite IFNB therapy, patients with ongoing disease activity which included new active lesions on MRI, relapse, or EDSS progression at the early stage, appeared to significantly increase the chance of suboptimal response at 3 years. The results of the present study suggest that both RS and MRS are useful for estimating the ensuing response to INFB in daily clinical practice in Korean MS patients, similar to those from Western countries [7,8].

While there was a considerable degree of heterogeneity within the definitions used, 16%- 29% of Western patients receiving IFNB showed ongoing disease activity in the first 2 years [15–17]. In the present study, 23% of patients were estimated as a high-risk group for suboptimal response using RS or MRS during early treatment with IFNB. This proportion of potential suboptimal responders at the early stage of treatment was consistent with previous Western studies, and the majority of these patients (86–92%) were found to show suboptimal responses over 3 years of IFNB therapy.

The sensitivity (75%) and specificity (> 96%) of RS and MRS confirmed their potential to predict suboptimal response to IFNB therapy. A previous study in patients with 4 years of follow-up from the initiation of treatment revealed low sensitivity (24%) but high specificity (97%) in MRS [8]. The higher sensitivity of RS and MRS in the current study may have been associated with the different definition of suboptimal response and timing of MRI monitoring compared to the previous study [8]. Previous studies with RS or MRS based on prospective research cohorts unified the follow-up duration of MRI scans as 6 or 12 months [7,8]. However, in this real-world setting, unified follow-up of MRI was difficult in individual patients, and serial MRI may often be compared using different protocols. Despite this limitation, the present study indicated that both scales were useful for predicting individuals at high-risk of a suboptimal response to IFNB in daily clinical practice.

On the other hand, 4 (7%) out of 56 patients who were considered at low-risk (score 0 or 1) by RS and MRS during early treatment ultimately had disease activity over 3 years of follow-up. To resolve this issue, Sormani and colleagues recently suggested that refining the scores may allow better prediction of treatment response [18]. Patients with a score of 1 estimated by MRS who were classified as being at intermediate risk, may require additional evaluation after 6 months; if the patient has then experienced ≥1 relapse in the additional 6 months or ≥2 new T2 lesions have appeared at the 6 month MRI scan, then patient can be associated with the high-risk group [18].

The major difference between RS and MRS is involvement of EDSS progression [8]. Some patients may show EDSS progression during early IFNB therapy in the absence of new active lesions on follow-up MRI scans and clinical relapse. Moreover, score 0 on RS is in line with the “no evidence of disease activity” or “freedom from disease activity” which has been recently considered as a potential ideal measure of the therapeutic responses [19]. In this study, the odds ratio for optimal responders who had RS of 0 was 9.4 (95% CI 2.4–37.5, p = 0.001). However, estimation of EDSS may not always be available in daily clinical practice and involve high inter-rater variability [20]. For these reasons, MRS was developed based on the recent observation that a combination of new active MRI lesions and relapses seem to be a surrogate for disability progression [8,16]. In the present study, RS and MRS independently predicted suboptimal responders and their scores were well matched. Thus, complementary use of these scores would facilitate detection of future suboptimal responders in daily practice.

This study had some methodological shortcomings due to its retrospective nature. Follow-up MRI scans of MS patients in early treatment with IFNB were not always performed in daily clinical practice; therefore, the number of the patients enrolled in this study was small. Therefore, ordinal logistic regression to analyze the odds of the various scores in predicting suboptimal response and also analyzing differences by baseline variables was not available. However, considering the 20–30 times lower prevalence of MS in Korea than those in Western countries, 70 cases could be comparable to a few hundreds cases in countries with higher MS prevalence [21,22].

In conclusion, evaluation of RS and/or MRS in daily clinical practice is useful to predict the response to INFB therapy and therefore would be helpful to optimize MS therapy.
